# OLDER ADULTS’ UNDERSTANDING OF SIGNALS COMMUNICATED BY ROBOT COMPANIONS FOR CAREGIVING

**DOI:** 10.1093/geroni/igad104.1985

**Published:** 2023-12-21

**Authors:** Bertram Malle, Vivienne Chi, Claudia B Rebola

**Affiliations:** Brown University, Providence, Rhode Island, United States; Brown University, Providence, Rhode Island, United States; University of Cincinnati, Cincinnati, Ohio, United States

## Abstract

Robotic animal-like companions for older adults are promising technologies to address companionship and new models of care. Our project, Affordable Robotic Intelligence for Elderly Support, aims to design smart communication capabilities in an Ageless Innovation Joy for All™ robotic cat. The cat robot with enhanced capabilities uses intuitive digital cues, including display screen, sound, and LED to create affordances to communicate various interaction intents to engage users into desirable actions. We conducted a study with 315 participants to understand the efficacy of designed affordances. A total of 8 affordances for 8 task scenarios, each with 3 variations of complete or incomplete information, simulating possible experiences were tested with users. Participants were randomly assigned to the conditions “full” (=display+sound+LED), “no_display” (=sound+LED), and “no_sound” (=display+LED) and were asked to provide a response interpretation of the experience signals and rank a list of potential interpretations in order of possibility. Results indicate that participants in the full experience group liked the robot the most (M=6.95 on a scale of 1-10) and gave the highest probability of the robot being a pleasant companion to older adults (M=7.17), compared to the no_display group (M_likability=6.01; M_companionship=6.58) and the no_sound group (M_likability=6.35; M_companionship=6.96). Participants in “no_display” had the greatest difficulty at inferring the robot’s intent. However, the no_sound group had less difficulty understanding the robot than the “full” experience group. Sound (e.g., meowing) appeared to be ambiguous, and removing it improved the communicability of the designed interface of this robot companion.

